# Thrombin antithrombin complex concentration as an early predictor of deep vein thrombosis after total hip arthroplasty and total knee arthroplasty

**DOI:** 10.1186/s12891-022-05532-1

**Published:** 2022-06-14

**Authors:** Zhencan Lin, Hao Sun, Deng Li, Zhiqing Cai, Meiyi Chen, Wenhui Zhang, Fangzhou Liu, Zhencheng Huang, Yimin Wang, Jie Xu, Ruofan Ma

**Affiliations:** 1 Department of Orthopedics, Sun Yat-sen Memorial Hospital, Sun Yat-sen University, Guangzhou, 510120 Guangdong China; 2 Department of Orthopedics, The Eighth Affiliated Hospital, Sun Yat-Sen University, Shenzhen, 518000 Guangdong China

**Keywords:** Total hip arthroplasty, Total knee arthroplasty, Deep vein thrombosis

## Abstract

**Aim:**

Early predictive markers of venous thromboembolism (VTE) after total hip arthroplasty (THA)/total knee arthroplasty (TKA) remain unclear. Our study identified early predictive markers for VTE after THA/TKA.

**Methods:**

A single-institution retrospective review study was conducted between May 2020 and April 2022 (*n* = 256). All patients underwent Doppler ultrasounds exam in preoperation and seventh day after surgery. Deep vein thrombosis (DVT) was defined by Doppler ultrasound of the lower extremities, which revealed thrombosis. Thrombin-antithrombin complex (TAT), thrombomodulin (TM), and plasmin-antiplasmin complex (PIC) concentration were tested from each patient’s preoperative and postoperative days 1, 4, 7, 14. These values were then accessed via receiver operating characteristic (ROC) curve analysis and further quantified the level of this risk by concentration.

**Results:**

On postoperative day 1 (pod-1), all patients’ TAT and PIC concentrations were significantly higher than those preoperatively (*p* < 0.05). The levels of TAT and PIC in patients in the DVT group on pod-1 were significantly higher than those in the non-DVT group (*p* < 0.05). At pod-1, the TAT concentration for DVT patients was 49.47 ng/mL compared to 20.70 ng/mL for non-DVT patients, PIC was 3.72μg/mL compared to 1.65μg/mL. ROC curve analysis demonstrated that a TAT concentration of 24.3 ng/mL had a sensitivity of 87.9% and a specificity of 69.1%.

**Conclusion:**

TAT levels on pod-1 may predict DVT early after THA/TKA, which makes it possible for early intervention to decrease the incidence of DVT.

## Introduction

Venous thromboembolism (VTE) is a common complication associated with total knee arthroplasty (TKA) and total hip arthroplasty (THA) [1]. At least 14 days of anticoagulant prophylaxis and the best duration of approximately 35 days were needed in patients undergoing THA/TKA [2]. VTE includes deep vein thrombosis (DVT) and pulmonary embolism (PE) [3]. PE may become a fatal, life-threatening complication. In the presence of thromboprophylaxis, the rate of venous thromboembolism (VTE) in patients undergoing major orthopedic surgery has been reported. The rate of DVT occurs between 2.22 and 3.29%, PE occurs between 0.87 and 1.99%, and fatal PE occurs in 0.30% of cases in America and Europe [4, 5]. DVT occurs in 1.40%; PE occurs in 1.10% of cases in Asia [6, 7]. Despite the relatively low incidence of DVT, this result is because ultrasound was not routinely performed, only for symptomatic patients following major orthopedic surgery before hospital discharge [4]. In a routine ultrasound screening study, 26 patients (47%) had asymptomatic distal DVT after TKA [8], and 39 patients (9.6%) had asymptomatic DVT after THA [9]. Thus, asymptomatic DVT also needs to be identified early to avoid the development of PE.

Up to this date, there has been a lack of effective indicators or methods to predict or identify VTE earlier. Currently, either P-selectin, D-dimer, or thromboelastography (TEG) do not indicate the risk of thrombosis [10–14]. Similar findings were obtained (our unpublished results). Thus, finding a new reliable, affordable, and easily available method to predict DVT is urgently needed.

Recently, new coagulation-associated hematological tests have emerged, such as thrombomodulin (TM), thrombin-antithrombin complex (TAT), and plasmin-antiplasmin complex (PIC). TM is a transmembrane glycoprotein that is highly expressed in endothelial cells (ECs) [15]. Antithrombin, the most abundant natural anticoagulant, regulates coagulation by combining thrombin to form the TAT complex [16]. The PIC consists of plasmin and its main physiological inhibitor, α2-antiplasmin (α2-AP), which can inhibit the fibrinolysis effect [17]. However, these indicators have been less studied in THA/TKA to predict DVT risk early. This study aimed to attempt to apply these indicators to predict DVT early.

## Methods

We conducted a retrospective review of THA/TKA performed between May 2020 and April 2022 in our hospital. All patients underwent Doppler ultrasounds exam in preoperation and seventh day after surgery. This study included a total of 256 patients, of whom 33 were diagnosed with DVT and 223 were not using ultrasound on postoperative day 7 (pod-7). All patients were treated with low molecular heparin (enoxaparin sodium) 0.4 mL through subcutaneous injection at 12 h after the surgery. On pod-2, enoxaparin sodium was administered subcutaneously once daily at 0.4 ml before discharge. Patients were excluded if they had been diagnosed with DVT before surgery. Patients were also excluded if they had a malignant tumor, blood system disease, or hepatic dysfunction.

### Laboratory evaluations and diagnosis method

Venous blood samples were collected before surgery and on pod-1, pod-4, pod-7, and pod-14. The levels of TM, TAT, and PIC were detected using a fully automatic chemiluminescence immune analyzer (Sysmex HISCL-5000). All patients underwent Doppler ultrasound of the lower extremities to diagnose or rule out DVT before surgery and pod-7. The two groups were divided into the non-DVT and DVT groups. The lower extremity deep veins studied included the external iliac vein, femoral vein, popliteal vein, posterior tibial vein, anterior tibial vein, peroneal vein, and muscular calf vein.

### Statistical analysis

Statistical analysis was performed with SPSS statistics 25.0 (SPSS, version 25.0) and MedCalc Statistical Software. The non-parametric test was performed to compare the two groups. Receiver operating characteristic (ROC) curves were plotted, and the area under the curve (AUC) was calculated to assess the specificity and sensitivity of distinguishing patients with and without DVT.

## Results

**Fig. 1:**
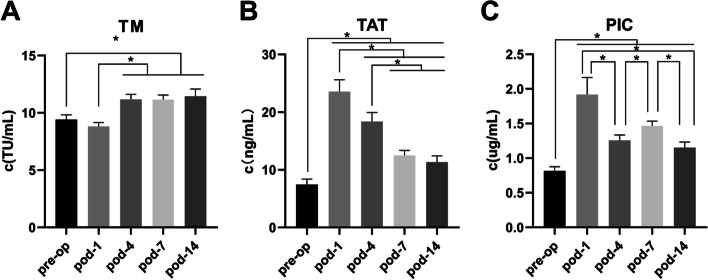
The change of TM, TAT, PIC in the perioperative. **A** TM. **B** TAT. **C** PIC. *Statistical difference was found between two groups. Bars represent 95% confidence interval. Pre-op, preoperative; pod, postoperative day; TM, thrombomodulin; TAT, thrombin-antithrombin complex; PIC, plasmin-antiplasmin complex

There were no significant differences between the two groups regarding age, sex, or body mass index (Table 1). For all cases, the values of TM, TAT, and PIC were not consistent during perioperative for all THA/TKA patients (Fig. 1). In our study, most of our patients were observed drastic activation of the clotting system after surgery for the TAT and PIC (Fig. 1). Statistically significant differences were found between preoperation and pod-1 in TAT and PIC. All of the others significant differences are based on statistical analyses shown in Fig. 1.

**Table 1:**
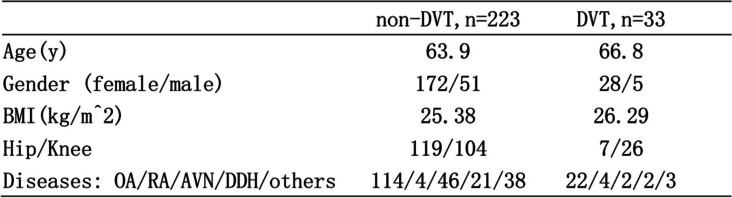
Demographics

*Abbreviations*: *BMI* Body mass index, *OA* Osteoarthritis, *RA* Rheumatic arthritis, *AVN* Avascular necrosis, *DDH* Developmental dysplasia of the hip

**Fig. 2:**
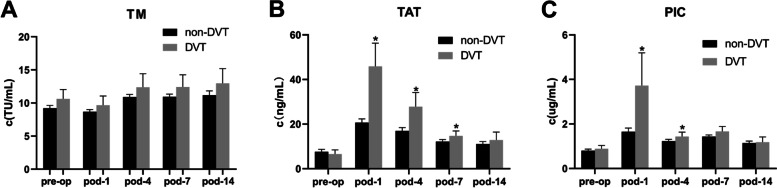
The non-DVT group compared to the DVT group in the perioperative. **A** TM. **B** TAT. **C** PIC. Bars represent 95% confidence interval. *Statistical difference was found between the non-DVT group and the DVT group at the same stage (*p* < 0.05)

Consequently, We focus on the value of TAT and PIC on pod-1. To further assess these findings, we conducted subgroup analyses of DVT events. There were no statistically significant differences at any time point between the two groups for TM. The levels of TAT and PIC in patients in the DVT group on pod-1 were significantly higher than those in the non-DVT group (*p* < 0.05). The level of TAT in the DVT group on pod-4, 7 was significantly higher than that in the non-DVT group (*p* < 0.05) (Fig. 2). A significant statistical difference was found between the two groups, both TAT and PIC, at the same time only in pod-1. Thus, we chose TAT and PIC data in pod-1 for ROC analysis.

**Fig. 3:**
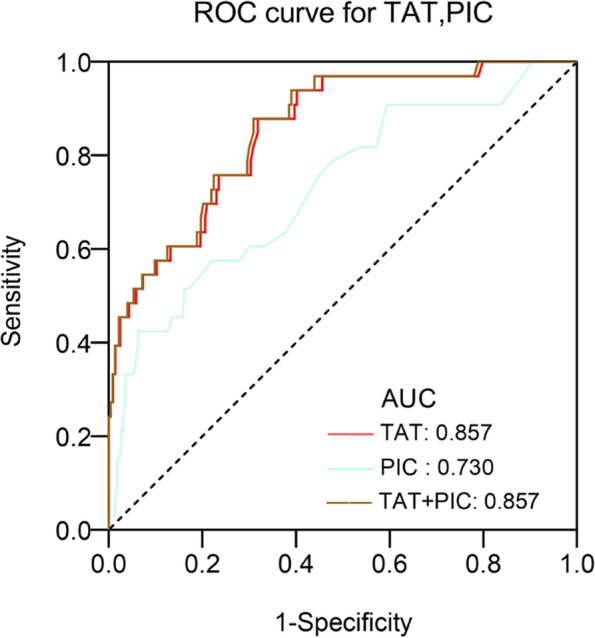
ROC curve analysis of biomarkers for the prediction of DVT. ROC, receiver operating characteristic curve; AUC, area under the curve; TAT, thrombin-antithrombin complex; PIC, plasmin-antiplasmin complex

According to ROC analysis (Fig. 3, Table 2), on pod-1, the optimal TAT cut-off value for thrombosis was 24.3 ng/mL, area under the ROC curve (AUC), 0.857 (95% CI, 0.791–0.922), with the highest Youden index (0.57), a sensitivity of 87.9% and a specificity of 69.1%; the optimal PIC cut-off value for thrombosis was 3.65 μg/mL, AUC, 0.730 (95% CI, 0.633–0.827), with the highest Youden index (0.36), a sensitivity of 42.4% and a specificity of 93.7%. Further analysis revealed that the plot combining ROC (TAT and PIC) had a similar area (0.857) under the ROC curve and odds ratio (OR). At the same time, AUC differences between TAT and TAT/PIC did not show statistical significance.

**Table 2:**
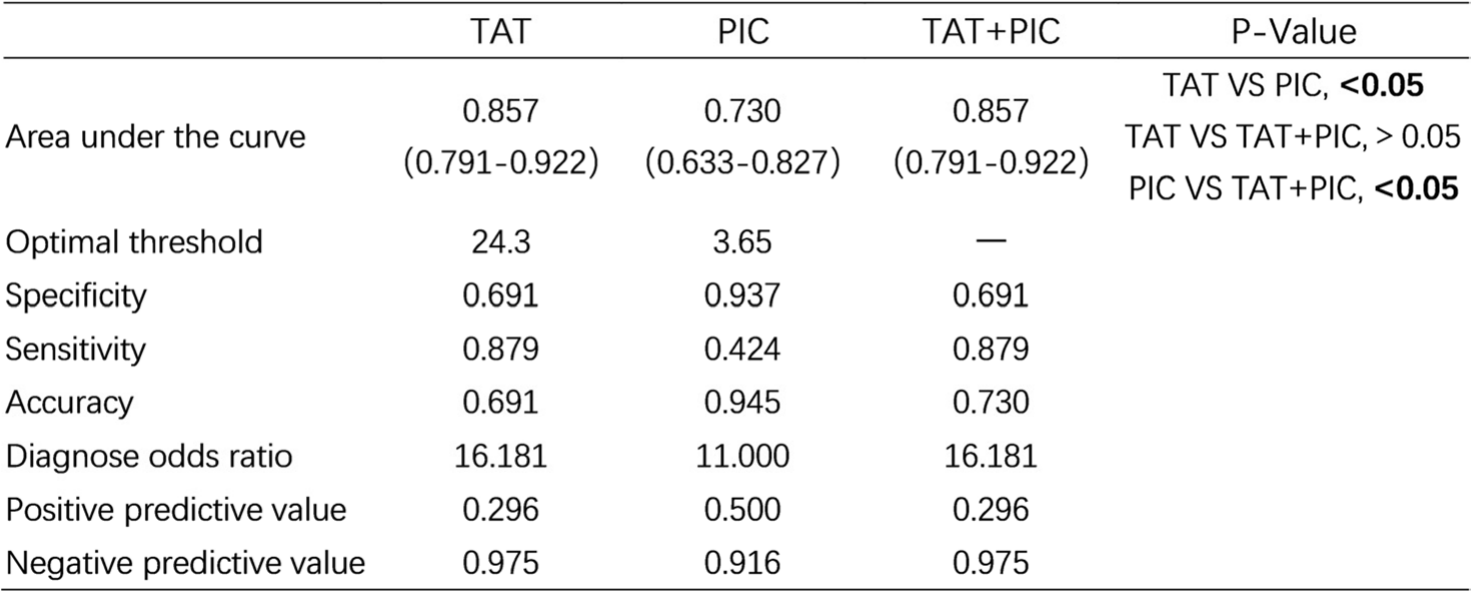
Receiver of TAT and PIC in the prediction of DVT

Bold value indicates statistically significant

## Discussion

DVT remains serious and common clinical complication worldwide after THK/TKA [18, 19]. It involves three risk factors: venous stasis, vascular injury, and hypercoagulability [20]. THA/TKA surgery results in significant hematological changes, tourniquet use, and reduced perioperative mobility, which elevates the risk of DVT. Once the thrombus is formed, there will be some symptoms, such as lower limb pain and swelling. To make matters worse, a thrombus may cause fatal PE.

Currently, most available diagnostic tests, such as P-selectin, D-dimer, and TEG, cannot predict the risk of thrombosis after THA/TKA. Thomas et al. determined that at 6 weeks after the operation, 92% of THA patient and 100% of TKA patients had serum D-dimer measurements higher than the institutional threshold (0.40 mg/mL) for a “positive” quantitative test; thus, D-dimer was not considered applicable to predict DVT event [10]. Shi et al. found that increased P-selectin levels were detected in the postoperative group. However, compared to the non-DVT group, there was no increase in the DVT group after THA [12]. Tareq et al. reported a statistically significant difference between VTE-positive and VTE-negative patients using TEG [13]. Thus, we attempted to find an economical, accurate, and simple way to predict the risk of thrombosis. In this study, we found a statistically significant difference between DVT patients and normal patients in TAT and PIC on pod-1. The ROC curve analysis showed that the levels of TAT on pod-1 may predict DVT early after THA/TKA.

In our study, the incidence rate of asymptomatic DVT was 12.89%(33/256). In the study by Vincent et al., the incidence of DVT was 12.8% after THA/TKA [21], similar to a study by Wu et al.; the rate was 9.6% [9]. These findings are consistent with our research. We found that TAT and PIC could sensitively reflect changes in coagulation status. TAT levels may be associated with VTE incidence [22]. Kobayashi et al. reported significant differences in the TAT levels between the DVT group and the normal group after medial opening-wedge high tibial osteotomy (OWHTO) [23]. Increasing levels of TAT may be an independent risk factor for VTE development in hospitalized Japanese patients receiving chemotherapy for malignancies [24]. However, currently, there are no studies regarding the use of TAT to predict DVT after THA/TKA. Moreover, none of the included studies were specific to the prediction of DVT early after THA/TKA. Our study found that the levels of TAT and PIC on pod-1 may be able to predict which patients progress to detectable DVT early after THA/TKA. More importantly, we need a higher sensitivity rather than specificity. Hence, it was indicated that merely using TAT was an independent prognostic marker and had a higher prognostic value compared with PIC. We established a cut-off value greater than 24.3 ng/mL for early prediction of a higher risk of thrombosis by ROC curve analysis, which is of great significance for the early prevention and treatment of DVT and reduce the incidence of PE during the perioperative period.

To improve risk stratification, we stratified the data according to the concentration levels of TAT (Table 3). Then, we calculated the positive predictive value (PPV) (positive = thrombosis) under stratification. The stratification of TAT has important clinical implications for predicting the risk of thrombosis. The TAT concentration on pod-1 correlated with the onset of DVT. According to the consequence, when TAT was > 30 ng/mL, the PPV was 0.3333. When TAT was ≥40 ng/mL, the PPV was 0.4500.

**Table 3:**
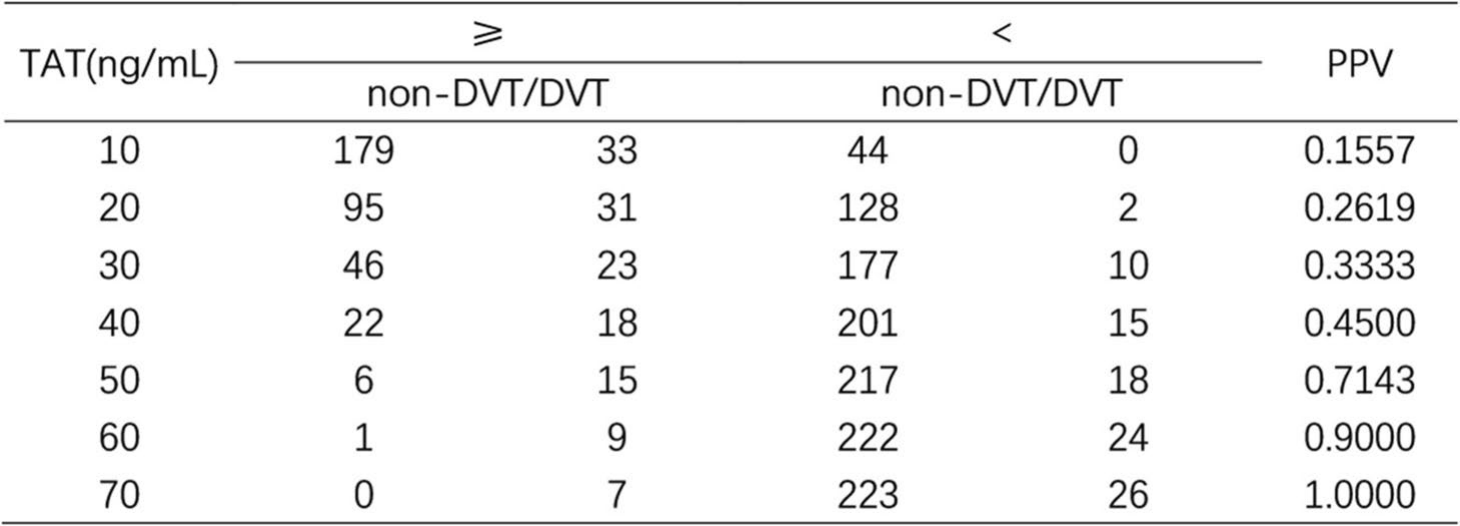
Data by concentration levels of TAT, calculating the PPV of DVT

*PPV* Positive predictive value

The stratified analysis for TAT showed that the TAT concentration increased as the risk of DVT increased. According to our health center’s condition, when TAT ≥30 ng/mL, we should focus on pain and swelling in the affected limb or multiple screen venous ultrasound testing of the lower extremity to identify thrombotic events earlier.

There are some limitations to this study. First, this was a single-center, retrospective study. Second, the sample size of this study was relatively small. Thus, we will enlarge the sample size of our study for further research. Multi-center prospective studies and large sample clinical studies are needed to illustrate and predict DVT in the future.

In summary, this study demonstrated that TAT has a high sensitivity but low specificity for the prediction of DVT. These results suggest that the levels of TAT on pod-1 may predict the occurrence of DVT. This study indicated that single indicator prediction was not inferior to combined indicators. Compared to other methods, a single indicator and single time point detecting TAT to predict DVT is simple, convenient, accurate, and effective.

## Data Availability

The data are available for download at https://figshare.com/articles/dataset/Untitled_Item/17119088.
